# Prognosis Prediction in Head and Neck Squamous Cell Carcinoma by Radiomics and Clinical Information

**DOI:** 10.3390/biomedicines12081646

**Published:** 2024-07-24

**Authors:** Shing-Yau Tam, Fuk-Hay Tang, Mei-Yu Chan, Hiu-Ching Lai, Shing Cheung

**Affiliations:** School of Medical and Health Sciences, Tung Wah College, Hong Kong

**Keywords:** HNSCC, HPV, machine learning, prognosis prediction, PWEM, radiomics, VEML

## Abstract

(1) Background: head and neck squamous cell carcinoma (HNSCC) is a common cancer whose prognosis is affected by its heterogeneous nature. We aim to predict 5-year overall survival in HNSCC radiotherapy (RT) patients by integrating radiomic and clinical information in machine-learning models; (2) Methods: HNSCC radiotherapy planning computed tomography (CT) images with RT structures were obtained from The Cancer Imaging Archive. Radiomic features and clinical data were independently analyzed by five machine-learning algorithms. The results were enhanced through a voted ensembled approach. Subsequently, a probability-weighted enhanced model (PWEM) was generated by incorporating both models; (3) Results: a total of 299 cases were included in the analysis. By receiver operating characteristic (ROC) curve analysis, PWEM achieved an area under the curve (AUC) of 0.86, which outperformed both radiomic and clinical factor models. Mean decrease accuracy, mean decrease Gini, and a chi-square test identified T stage, age, and disease site as the most important clinical factors in prognosis prediction; (4) Conclusions: our radiomic–clinical combined model revealed superior performance when compared to radiomic and clinical factor models alone. Further prospective research with a larger sample size is warranted to implement the model for clinical use.

## 1. Introduction

Head and neck squamous cell carcinoma (HNSCC) is the seventh most prevalent cancer worldwide, with about 890,000 new cases and 450,000 mortalities in 2020 [1]. HNSCCs occur in a wide range of primary sites in the head and neck region, ranging from the oral cavity to the pharynx. Multi-model treatments are used as the first-line treatment for HNSCC patients, including surgery, radiotherapy, chemotherapy, and immunotherapy, depending on the particular tumor position and staging [2]. Radiotherapy is the elective treatment for most HNSCC cases when the cancer is unresectable at a locally advanced stage [3]. Despite the advancement of multi-modality treatment, the 5-year survival rate in patients with HNSCC is still less than 50% due to late diagnosis and the high risk of disease recurrence [4].

One important feature of HNSCCs is their high heterogeneity. This consists of anatomical, biological, and molecular heterogeneities [5]. This affects the treatment outcome among patients treated with the same standard therapy [6]. This property undermines the development of effective biomarkers and the effectiveness of conventional tumor biopsy [6]. To date, only programmed death-ligand 1 (PD-L1) and human papillomavirus (HPV) expressions are considered as useful biomarkers in HNSCC [7]. To tackle this situation, radiomics has been suggested for the further development of personalized treatment of HNSCC [6].

Radiomics consists of extracting quantitative information from medical images, and associating it with clinical features to construct models for prognosis prediction with different machine-learning algorithms [8]. Radiomics can potentially identify previously unknown tumor markers to improve prognosis prediction in large datasets [9]. Due to its ability to detect tumor heterogeneity by extracting and analyzing sub-visual features from various imaging modalities, radiomics has been commonly studied in relation to HNSCC for outcome prediction with promising results [10,11,12].

Different machine-learning algorithms have their strengths and limitations. It is suggested that by combining the predictions from multiple machine-learning algorithms, a more reliable prediction will be achieved by averaging and mitigating their limitations [13]. Long et al. [14] employed an ensembled machine-learning algorithm to predict survival in patients with hepatocellular carcinoma (HCC) and bone metastasis. Their ensemble model demonstrated the best performance as compared to individual machine-learning algorithms, with an area under curve (AUC) of 0.779. This hints that the ensemble technique can potentially improve prognosis prediction and facilitate clinical decision-making.

Apart from radiomic data, clinical information including tumor staging and patient demographics provides valuable information for prognosis prediction. It is suggested that combining radiomic and clinical features will produce a synergistic effect, which enhances predictive performance and accuracy. Gangil et al. [15] compared the predictive capabilities of machine-learning algorithms using clinical, radiomic, and radiomic–clinical datasets in HNSCC. They revealed that the model that combined radiomic–clinical datasets exhibited superior predictive power in comparison to the model which relied solely on clinical features. Meanwhile, the model constructed with radiomic features alone demonstrated poor performance in predicting clinical outcomes.

The ensemble technique may be combined with radiomic and clinical information for further enhancing the predictive outcome. Tang et al. [16] integrated radiomic data obtained from radiotherapy (RT) planning CT with clinical information for prognosis prediction in patients with non-small cell lung cancer (NSCLC). Radiomic and clinical features were first studied by five machine-learning algorithms with a voted ensemble machine-learning (VEML) model. Then, a probability-weighted strategy was used to incorporate radiomic and clinical features. The results showed that the combined model had superior performance compared to the radiomic model. This demonstrated that the combined model possesses the ability to improve prognosis prediction.

Since HPV status has been recognized as an important prognostic biomarker in HNSCC with a strong link to oropharyngeal carcinoma (OPC), there are some studies that combined radiomic and HPV status for a prognosis prediction. Wang et al. [17] combined radiomic features and HPV status to perform a risk classification in patients with OPC. Meanwhile, Ou et al. [18] showed that combining HPV p16 status and radiomics could outperform models using p16 status or radiomics alone in locally advanced HNSCC, with the majority of cases being OPC (68%). Therefore, the addition of HPV status information could be important for a prognosis prediction of HNSCC.

Heterogeneity remains a major concern affecting HNSCC prognosis and HPV information has been emerging as an important biomarker in HNSCC. Meanwhile, the use of an ensembled technique with both radiomic and clinical information may offer excellent predictive capability compared to previous studies. In this study, we aimed to predict 5-year overall survival in HNSCC radiotherapy patients by integrating radiomic and clinical information in machine-learning models.

## 2. Materials and Methods

### 2.1. Data Acquisition

The datasets consisting of treatment planning CT and radiotherapy (RT) structures sets were collected from The Cancer Imaging Archive (TCIA). This is an openly accessible database providing collections of medical images from various imaging modalities and is regulated by the Frederick National Laboratory for Cancer Research.

With permission granted from TCIA, a total of 627 datasets of HNSCC patients receiving radiotherapy at MD Anderson Cancer Center were acquired from TCIA’s ‘HNSCC’ collection [19]. The collection comprised head and neck cancer patients receiving radical radiotherapy from 2003 to 2013, and oropharyngeal cancer patients receiving radiotherapy between 2005 and 2012. Pre-treatment planning CT images, along with RT structures and gross tumor volumes (GTV) contoured by professional clinical oncologists in Digital Imaging and Communications in Medicine (DICOM) format were obtained from the datasets. Furthermore, patient demographic and pathological information, including gender, age, smoking status, diagnostic site, tumor stage, HPV status, treatment modality, and 5-year overall survival status were also collected.

### 2.2. Study Workflow

Patients who satisfied the following requirements were included in the study: (1) they underwent treatment planning CT with gross tumor volume (GTV) delineated by clinical oncologists, (2) they possessed complete pathologic information including HPV status, and (3) they had a definite tumor staging. Initially, datasets comprising CT images, delineated RT structures, and clinical information were collected from TCIA. After that, radiomic feature extraction was conducted, and these features were subsequently inputted into the predictive models.

In this study, the primary endpoint was defined as 5-year overall survival (OS). To minimize the potential bias caused by imbalanced data and reduce the risk of overfitting, a balanced sample consisting of the same number of individuals who were alive and dead 5 years after diagnosis was employed by random selection. Then, the selected sample datasets were randomized to minimize selection bias and reduce the impact of confounding variables. Eventually, the data were analyzed using 5 machine-learning algorithms. The process from sample balancing was repeated 5 times to ensure that all samples were studied at least once. The outcomes of each iteration were then averaged to obtain more reliable results.

### 2.3. Feature Extraction

The extraction of radiomic features from GTV was employed utilizing the PyRadiomics extension in 3D Slicer software (v. 4.10.2), developed by the Computational Imaging and Bioinformatics Lab at Harvard Medical School [20,21]. The predictive model was developed by extracting 107 radiomic features from the planning CT images. These radiomic features include tumor shape, gray-level co-occurrence matrix, gray-level dependence matrix, first-order statistics, gray-level size zone matrix, gray-level run length matrix, and neighboring gray-tone difference matrix features [21]. Subsequently, the extracted features were analyzed by 5 machine-learning algorithms utilizing R software (v. 4.1.3) to predict the prognosis of HNSCC.

### 2.4. Machine Learning

**Figure 1:**
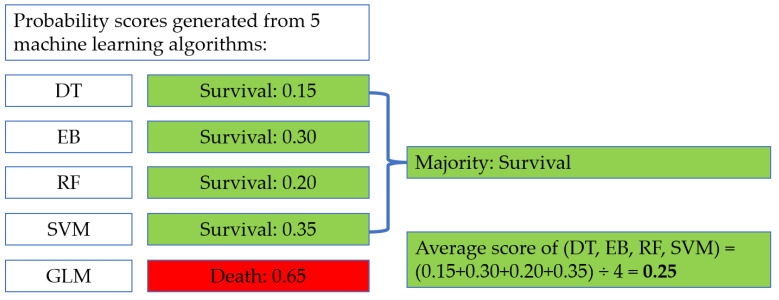
Procedure of VEML score calculation.

Five common machine-learning algorithms were utilized in this study, including decision tree (DT), extreme boost (EB), random forest (RF), support vector machine (SVM), and generalized linear model (GLM) algorithms. The brief introduction of the 5 machine-learning algorithms is summarized in Table 1. For each algorithm, the targeted population was randomly divided into three cohorts. 70% of the samples were inputted into a training cohort to establish patterns, whereas both the validation cohort and the testing cohort contained 15% of the data. To enhance the accuracy of predictive performance, voted ensemble machine learning (VEML) was then employed by incorporating the probability scores generated from 5 algorithms to achieve a more realistic prediction when there were conflicts occurring between models. VEML was employed in our previous publication [16] and the feature is summarized in Figure 1.

### 2.5. Probability-Weighted Enhanced Model (PWEM)

Our previous studies have indicated that integrating radiomic features with clinical factors could enhance the accuracy of predictive models of NSCLC [16]. Moreover, patient demographic and pathological information also had a satisfactory performance in prognosis prediction of HNSCC [18,23,24]. To improve the predictive performance of the VEML model, we combined the results of VEML of radiomic and clinical factors by a probability-weighted approach.

**Figure 2:**
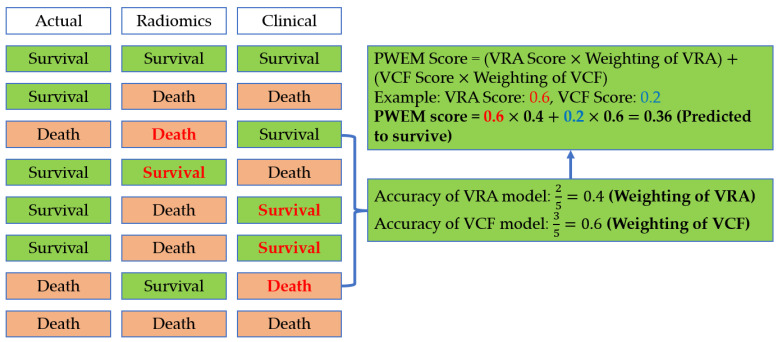
Procedure of PWEM score calculation.

The PWEM was illustrated in our previous study [16]. Briefly, the model comprised both hard voting and soft voting techniques (Figure 2). For hard voting, a VEML model was utilized to generate a VEML score for the radiomic model and the clinical factor model. These VEML scores represented the estimation and likelihood of the survival outcome from solely considering radiomic (VRA) or clinical factors (VCF). For soft voting, a probability-weighted enhanced approach was employed to assign each model’s weighting based on their respective ability to predict prognosis in the validation cohort. Predictive weighting is the factor that reflects the model’s probability of acquiring a correct prediction under a conflicting situation. The weighting of each model is counted according to the probability of getting a correct prediction by each model among the conflicted predictions. By multiplying the VEML score of each model to its respective predictive weighting, the sum of two models would be used as the final score ranging from 0 to 1. A score lower than 0.5 suggests that the patient is likely to survive at the study endpoint. Meanwhile, a weighted score of 0.5 or higher indicates mortality prediction at the study endpoint.

The weighted score was determined by using the following equation [16]:

PWEM Score = (VRA Score × Weighting of VRA) + (VCF Score × Weighting of VCF)

### 2.6. Data Analysis

The descriptive data were presented as mean ± standard deviation. To assess the predictive performance of the radiomic and clinical factor model using a single machine-learning algorithm, VEML and PWEM, the receiver operating characteristic (ROC) curve was utilized to demonstrate the prognostic performance of the models in various metrics, including area under the curve (AUC), accuracy, sensitivity, and specificity. Moreover, for the clinical factor model using a random forest algorithm, the mean decrease accuracy and mean decrease Gini were used to assess the importance of each clinical factor in prognosis prediction. Additional evaluations by chi-square tests were employed to confirm the significance of clinical factors in HNSCC survival. A *p* value of less than 0.05 is considered as statistically significant for this study.

## 3. Results

### 3.1. Patient Demographics

The dataset consists of 627 patients diagnosed with HNSCC. Out of 627 datasets obtained, 309 cases with missing data in their HPV status were excluded from this study. Eighteen cases with missing data in radiotherapy structures were also excluded. Additionally, one case was excluded due to unknown smoking status. A total of 299 cases were eventually included in this study (Table 2). For the included subjects, 238 subjects were alive at the study endpoint while 61 were dead. Their diseases were staged by the TNM system of the American Joint Committee on Cancer (AJCC), with a majority of patients (81%) who were diagnosed with stage IV disease. The database comprises 84% male and 16% female with a median age of 57. The majority of patients (88%) were detected positive with HPV infection.

### 3.2. Predictive Performance of Individual Machine-Learning Algorithm

Five machine-learning algorithms were used in this study on radiomic and clinical information. In terms of the average predictive performance for different machine-learning algorithms, the RF algorithm demonstrated the best results across various metrics in radiomic and clinical results (Table 3). Among the five randomized results, the RF model achieved the highest AUC values in both radiomic and clinical results of 0.79 and 0.76, respectively. For sensitivity, the RF model performed the best in the radiomic model while GLM performed the best in the clinical model. The RF model also achieved highest accuracy while SVM achieved the highest specificity.

### 3.3. Performance Evaluation for VEML Models and PWEM

**Figure 3:**
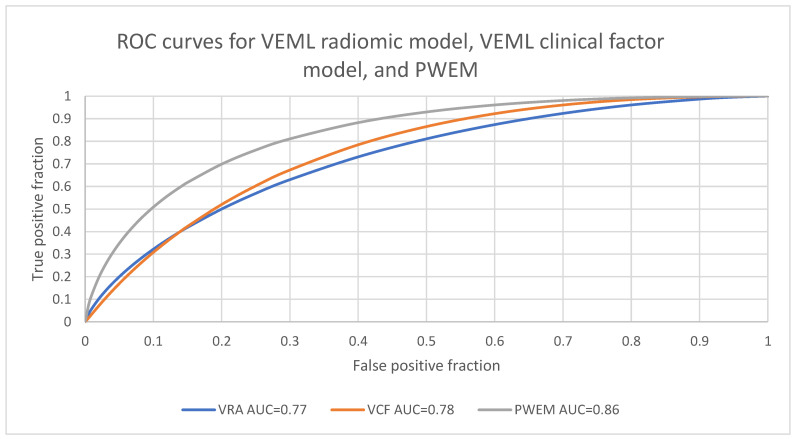
ROC curves of VEML models and PWEM.

Regarding the predictive performance for the VEML radiomic (VRA) model, the VEML clinical factor (VCF) model, and PWEM in patients’ overall survival, they attained AUCs of 0.77, 0.78, and 0.86, respectively (Figure 3). The highest levels of sensitivity, specificity, and accuracy were achieved by PWEM, with values of 0.73, 0.82 and 0.76, respectively. The VRA model demonstrated slightly higher sensitivity, specificity, and accuracy compared to the VCF model (Table 4). For the comparison in accuracy, PWEM was significantly higher than the VCF model by the Kruskal–Wallis test (*p* = 0.031). There were no significant differences in the AUC, sensitivity, and specificity between the three models.

### 3.4. Significance for Individual Clinical Factor

For the clinical factor model using the RF algorithm, the mean decrease accuracy (MDA) and mean decrease Gini (MDG) are listed in Table 5. For MDA, only the T stage has an MDA value of significantly greater than zero from the one-sample Wilcoxon test results (average MDA: 7.41, *p* = 0.043). While for MDG, the Kruskal–Wallis test showed that T stage, age, and disease site were significantly greater than other factors (*p* < 0.001). The post-hoc Dunn test showed that the MDA of the T stage is significantly greater than the use of surgery, the use of chemotherapy, and gender, while age was significantly greater than the three mentioned factors and HPV status. In addition, disease site was significantly greater than use of surgery.

The chi-square test was employed to determine the presence of statistically significant associations between clinical factor and survival outcome (Table 6). The only significant association was found on T stage ($x^{2}$ = 21.53, *p* = 0.0002), where T1 and T2 have significantly more survival cases than T4 in Bonferroni-adjusted pairwise comparisons.

## 4. Discussion

### 4.1. Performance in Machine-Learning Algorithms

In our study, the data were randomly selected to have the same number of survival and death cases in each comparison to reduce the risk of overfitting. The selected data were further randomized five times before the running of machine-learning algorithms. The random selections of cases were performed five times, and all the included cases were selected for study at least once. This approach minimizes selection bias, random fluctuation during running of the machine-learning algorithm, and ensures good representation of all the 299 included cases in the study results.

From the results of using a single machine-learning algorithm, the RF algorithm performed generally the best among the five machine-learning algorithms in both radiomic and clinical factor models. The RF model employs an ensemble technique that incorporates the results of various decision trees to generate a consolidated outcome. It randomly selects and observes features from a dataset, creating a collection of decision trees that guide decision making. This approach allows RF models to achieve superior predictive performance compared to most other machine-learning models [25]. For the use of VEML approach in both radiomic (VRA) and clinical factor (VCF) models, no significant improvement could be found in AUC, sensitivity, specificity, and accuracy when compared with the RF model. The possible reason for this is that the DT and GLM were performing poorly in both radiomic and clinical factor models. Therefore, the VEML approach could not mitigate most of the wrong predictions from the RF model, leading to a similar predictive performance as the RF model.

### 4.2. Importance of PWEM

When we utilized both radiomic features and clinical factors with PWEM, the predictive performance on HNSCC prognosis could be improved. The PWEM achieved an AUC of 0.86 with the accuracy significantly greater than the VCF model. This highlights the complementary nature of radiomic and clinical factors, resulting in a more reliable and precise prognosis prediction.

A comparable previous study by Mes et al. [26] combined radiomic signature, clinical characteristics, and HPV status through Cox regression to predict overall survival in HNSCC patients. Their combined model revealed an AUC of 0.75 and 0.81 in oral cavity and oropharyngeal disease, respectively. Ger et al. [27] proposed a model using multivariate Cox regression to determine the survival of patients with HNSCC and acquired AUCs between 0.72–0.73 with radiomic features extracted from contrast CT images and PET images. Meanwhile, Alfieri et al. [28] utilized the radiomic model of least absolute shrinkage and selection operator (LASSO) with logistic regression to examine the prognostic role of MRI radiomic features, obtaining AUCs ranging from 0.78 to 0.83. As PWEM considers radiomic and clinical features as independent variables with distinct natures, they were separately analyzed in machine learning [16]. By assigning greater weights to the more important classifiers, the PWEM can achieve higher accuracy [29]. Therefore, our prediction model of PWEM achieved a good AUC of 0.86 in predicting HNSCC prognosis with slightly better performance than previous studies. It is anticipated that PWEM could be further investigated for HNSCC prognosis prediction in clinical environments.

### 4.3. Clinical Factors as Important Prognostic Markers

We noticed that several clinical factors are more important for prognosis prediction using MDA, MDG, and chi-square tests. These factors include T stage, age, and disease site. When compared with previous studies, Howard et al. [30] utilized machine-learning algorithms to predict survival in HNSCC patients and evaluated the association between clinical factors and model accuracy. They revealed that age is the most significant factor for accurate prediction, following by years of diagnosis, tumor (T) stage, HPV status, and primary sites. Meanwhile, Kotevski et al. [31] investigated the role of machine learning in predicting 2-year cancer-specific survival (CSS) in patients with HNSCC. They observed that higher stage, T3 and T4 classification, and hypopharyngeal tumors were associated with poor prognosis. However, due to largely missing data regarding HPV information, it was excluded from their analysis.

HPV status is recognized as a significant prognostic biomarker in HNSCC. Tumors that test positive for HPV exhibit distinct characteristics and are highly vulnerable to radiotherapy and chemotherapy, leading to a more favorable prognosis as compared to tumors with negative HPV status [32]. While for our chi-square results ($x^{2}$ = 3.76, *p* = 0.052), HPV status was found to be a marginally insignificant factor in prognosis with an observed survival rate of 81.3% and 67.6% for HPV-positive and HPV-negative cases, respectively. The MDA and MDG analyses also showed that HPV status was not among the important clinical factors for prognosis prediction. The reason for this is that our findings could be affected by skewing of data as most of the selective patients belonged to stage 4 and HPV-positive groups. Nevertheless, based on our findings, it is suggested that T stage, age, and disease site were crucial for prognosis prediction and should be considered for building future prediction models.

### 4.4. Future Development of Ensemble Machine Learning in HNSCC

Our study combined radiomic features and clinical information through a probability-weighted approach to predict prognosis in HNSCC patients. Since HNSCC is characterized by its extensive genomic profile, which leads to varying treatment response and prognosis among patients [6], it is suggested that the prediction model can be enhanced by integrating genomic information to guide risk classification and prognosis prediction. Spielvogel et al. [5] evaluated the prognostic value of radiogenomic biomarkers and resulted in a good performance with an AUC of 0.72. Therefore, the integration of genomic data into our current model may further improve the prognosis prediction.

### 4.5. Study Limitations

Although our study has achieved an outstanding predictive performance of HNSCC prognosis by the PWEM approach, there are some limitations in our study. First, the sample size is limited after excluding subjects with incomplete information. Among the 627 patients in the selected database, only 299 patients contained complete information and HPV status. Most of the selected cases were stage 4, oropharyngeal cancer site, and HPV-positive. This limits the generalizability of the findings. Second, the data are in retrospective format and the cases were collected between 2003 and 2013. The advancement of treatment modalities and target delineation may impact the prognosis of new cases. This may also affect the reliability of the findings. It is suggested that conducting a larger-scale prospective study of various stages and disease sites will better predict the prognostic outcome of HNSCC patients [33].

## 5. Conclusions

To conclude, we employed a probability-weighted approach integrating both radiomic and clinical information to predict HNSCC survival. Our radiomic–clinical combined model revealed superior performance (AUC = 0.86) when compared to radiomic and clinical factor models (AUC = 0.77 and 0.78, respectively) alone. It is evident that the ensemble model can improve prognosis prediction. Nevertheless, further prospective research with larger sample sizes is required to implement the model for clinical use. Furthermore, we revealed that T stage, age, and disease site were the most important prognostic factors in the clinical factor model.

## Figures and Tables

**Table 1:** Brief introduction of the 5 machine-learning algorithms [22].

Machine Learning Algorithm	Brief Introduction
Decision tree (DT)	A conditional tree algorithm with a recursive partitioning approach for data mining. It does not require normalization and scaling of data but is subject to the weakness of bias and variances.
Extreme boost (EB)	This has a weight with each observation in the dataset and builds a series of models of decision trees by boosting the weight to the model that incorrectly classified the observation. It can handle complex data with high predictive accuracy. However, it could be easily affected by overfitting.
Random forest (RF)	A collection of unpruned decision trees with available variable subsets. It is robust to noise, exhibiting less bias and variances than a single decision tree. However, it can suffer from overfitting and may lead to poor generalization on new data.
Support vector machine (SVM)	Identifies data at the boundaries between classes and identifies the line that separated the classes in prediction. It can handle high-dimensional data but is sensitive to noise and outliers.
Generalized linear model (GLM)	Fits a statistical model to data for a regression model in prediction. It can handle different target distributions but is sensitive to outliers.

**Table 2:** Summary of patient demographics and tumor characteristics.

Category		No. of Subjects (%)
Survival at endpoint	Yes	238 (80%)
	No	61 (20%)
Gender	Male	250 (84%)
	Female	49 (16%)
Age	<50	47 (16%)
	50–59	137 (46%)
	60–69	45 (15%)
	≥70	36 (12%)
Smoking status	Non-smoker	114 (38%)
	Ex-smoker	112 (37%)
	Current smoker	73 (25%)
Disease site	Base of tongue	144 (48%)
	Tonsil	119 (40%)
	Glossopharyngeal sulcus	9 (3%)
	Soft palate	4 (1%)
	Glottis	3 (1%)
	Oral cavity	2 (<1%)
	Hypopharynx	1 (<1%)
	Not otherwise specified	17 (6%)
Overall stage	I	4 (1%)
	II	8 (3%)
	III	46 (15%)
	IV	241 (81%)
T stage	Tis	1 (<1%)
	T1	73 (24%)
	T2	115 (38%)
	T3	66 (22%)
	T4	44 (15%)
N stage	N0	26 (9%)
	N1	36 (12%)
	N2	231 (77%)
	N3	6 (2%)
HPV status	Positive	262 (88%)
	Negative	37 (12%)
Use of surgery	Yes	289 (97%)
	No	10 (3%)
Use of chemotherapy	Yes	253 (85%)
	No	46 (15%)

**Table 3:** Predictive performance of individual machine-learning algorithm.

Machine-Learning Algorithm	Radiomic Model				Clinical Model			
	AUC	Sensitivity	Specificity	Accuracy	AUC	Sensitivity	Specificity	Accuracy
Decision tree (DT)	0.67 ± 0.08	0.68 ± 0.15	0.52 ± 0.23	0.62 ± 0.02	0.63 ± 0.08	0.55 ± 0.15	0.60 ± 0.14	0.56 ± 0.10
Extreme boost (EB)	0.73 ± 0.07	0.68 ± 0.08	0.74 ± 0.12	0.71 ± 0.07	0.74 ± 0.06	0.60 ± 0.11	0.67 ± 0.12	0.62 ± 0.02
Random forest (RF)	0.79 ± 0.08	0.70 ± 0.14	0.76 ± 0.15	0.72 ± 0.06	0.76 ± 0.05	0.61 ± 0.11	0.78 ± 0.14	0.67 ± 0.04
Support vector machine (SVM)	0.75 ± 0.08	0.57 ± 0.14	0.84 ± 0.11	0.67 ± 0.06	0.75 ± 0.06	0.54 ± 0.15	0.90 ± 0.10	0.66 ± 0.09
Generalized linear model (GLM)	0.51 ± 0.06	0.51 ± 0.12	0.55 ± 0.15	0.52 ± 0.06	0.71 ± 0.07	0.65 ± 0.10	0.65 ± 0.10	0.64 ± 0.07

**Table 4:** Predictive performance of VEML models and PWEM.

Predictive Model	AUC	Sensitivity	Specificity	Accuracy
VEML radiomic model (VRA)	0.77 ± 0.11	0.69 ± 0.13	0.76 ± 0.18	0.72 ± 0.06
VEML clinical factor model (VCF)	0.78 ± 0.05	0.60 ± 0.11	0.73 ± 0.10	0.64 ± 0.04
PWEM	0.86 ± 0.07	0.73 ± 0.15	0.82 ± 0.15	0.76 ± 0.08

**Table 5:** Mean decrease accuracy (MDA) and mean decrease Gini (MDG) of the clinical factor model using a random forest algorithm.

Clinical Factor	MDA	One-Sample Wilcoxon Test (MDA vs. 0)	MDG
T stage	7.41 ± 4.09	0.043	4.16 ± 0.51
Age	3.39 ± 3.98	0.14	4.34 ± 0.53
N stage	2.16 ± 2.75	0.14	1.86 ± 0.35
Use of surgery	1.47 ± 3.93	0.69	0.55 ± 0.25
HPV status	1.37 ± 2.99	0.50	1.01 ± 0.30
Disease site	0.63 ± 6.42	0.89	3.30 ± 0.28
Overall stage	0.62 ± 2.65	0.89	1.21 ± 0.20
Use of chemotherapy	−0.32 ± 2.41	0.89	0.87 ± 0.16
Gender	−1.26 ± 2.40	0.35	0.90 ± 0.23
Smoking status	−3.56 ± 2.31	0.043	2.25 ± 0.11

**Table 6:** Chi-square test for the association between clinical factor and survival outcome.

Clinical Factor	Chi-Square Statistics ($x^{2}$)	*p* Value
T stage	21.53	0.0002
Age	6.93	0.14
N stage	3.51	0.32
Use of surgery	0.59	0.44
HPV status	3.76	0.052
Disease site	9.94	0.19
Overall stage	3.64	0.30
Use of chemotherapy	0.060	0.81
Gender	0.15	0.70
Smoking status	1.13	0.57

## Data Availability

The data presented in this study are available in The Cancer Imaging Archive at https://doi.org/10.7937/k9/tcia.2020.a8sh-7363 (accessed on 1 June 2024), reference number HNSCC Version 4. These data were derived from the following resources available in the public domain: https://www.cancerimagingarchive.net/collection/hnscc/ (accessed on 1 June 2024).
